# Ovarian Dermoid (Mature Cystic Teratoma) in a Postmenopausal Woman: Incidence of Sonographic Signs

**DOI:** 10.7759/cureus.17581

**Published:** 2021-08-30

**Authors:** Ravikanth Reddy

**Affiliations:** 1 Radiodiagnosis, St. John's Hospital, Bengaluru, IND

**Keywords:** postmenopausal, complications, sonological signs, high resolution ultrasonography, mature cystic teratoma, ovarian dermoid

## Abstract

Ovarian dermoid is a common surgically treatable cause of female infertility. Although the fat component of mature cystic teratoma (MCT) appears hyperechoic on ultrasonography, sometimes it poses a diagnostic challenge to differentiate from a complex ovarian cyst / hemorrhagic cyst. The varied presentation of MCT on ultrasonography is due to varying proportions of components belonging to all three germ cell layers such as epithelium, hair, bone, tooth, and cartilage. This case report describes the high-resolution ultrasonography appearance of MCT in a 48-year-old nulliparous post-menopausal woman and provides an elaborative note on how reliable diagnostic signs of MCT on ultrasonography and prompt recognition of the entity has a favorable outcome on prognosis.

## Introduction

Mature cystic teratomas (MCTs) of the ovary comprise up to 10-25% of all ovarian neoplasms and are usually seen in the younger age groups [1]. MCTs are usually an incidental finding during routine imaging studies or pelvic surgeries often performed for other indications. MCTs are complicated by malignant degeneration in only 1-2% of cases [2]. Rarely, an MCT may cause complications such as hydroureteronephrosis, small bowel obstruction, or entero-ovarian fistula formation. Rupture of the ovarian dermoid with resultant chemical peritonitis is an acute complication that needs urgent surgical exploration. Virilizing ovarian dermoid cysts, which are exceedingly rare and often found in postmenopausal women. have also been reported. The most common adnexal mass lesion associated with ovarian torsion is ovarian dermoid [3]. Herein, we describe the sonographic appearances and radiological signs associated with MCT for reliable diagnosis of the entity in a resource-starved setting with ultrasonography as the only functioning imaging modality.

## Case presentation

**Figure 1:**
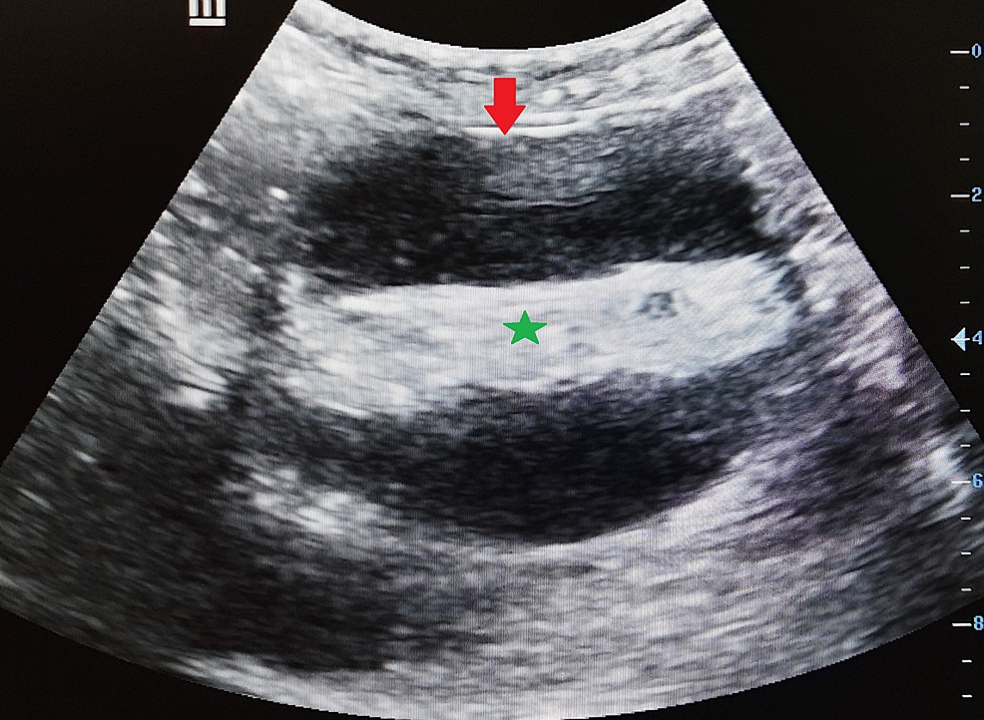
Transverse high-resolution ultrasonography image demonstrating a well-circumscribed cystic adnexal lesion (arrow) with evidence of a fat-fluid interface sign (star) demonstrating multiple coarse internal echoes causing posterior acoustic enhancement in a histopathologically proven case of atypical ovarian dermoid. Note the appearance, which is similar to a complex ovarian cyst.

A 48‐year‐old nulliparous postmenopausal woman with complaints of vague left-sided pelvic pain since four weeks attended the department of gynecology. She gave a history of infertility after being married for 18 years and had attained menopause three years prior. General physical examination was unremarkable. Systemic examination revealed tenderness in the left iliac fossa region. On high-resolution ultrasonography, there was a heteroechoic solid-cystic lesion arising from the left ovary measuring 5.9 x 4.7 cm (Figure 1). There was evidence of a fat-fluid interface sign with the lesion demonstrating multiple coarse internal echoes causing posterior acoustic enhancement.

**Figure 2:**
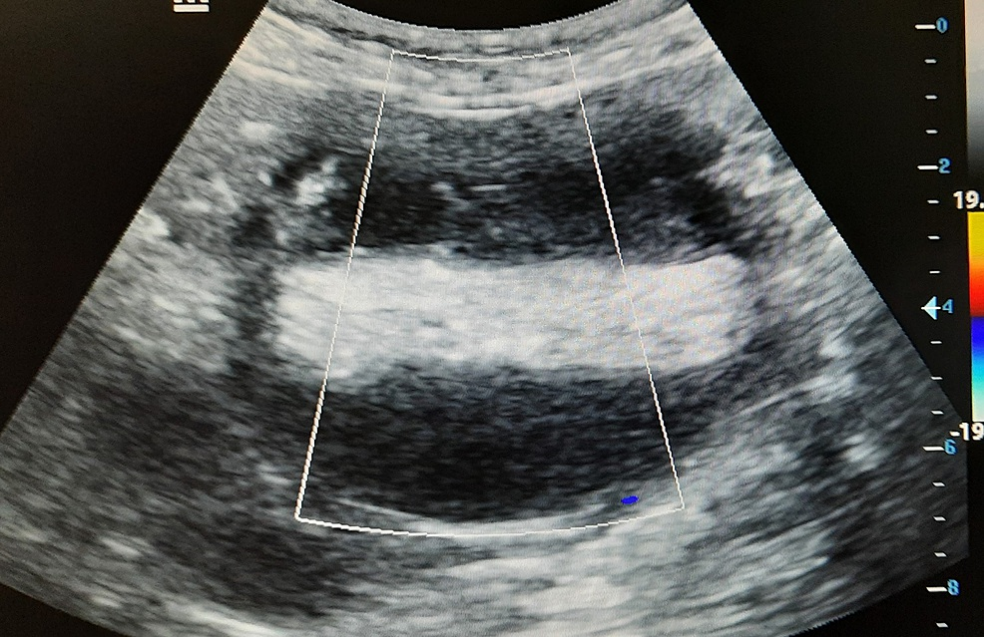
Color Doppler ultrasonography image demonstrating no internal vascularity within the heterogeneously appearing cystic adnexal lesion.

The heteroechoic adnexal cystic lesion did not demonstrate internal vascularity on color Doppler ultrasonography (Figure 2). The left ovary was not separately visualized from the lesion. Furthermore, there was no sonographic evidence to suggest torsion of the involved ovary. The findings were consistent with an ovarian dermoid (mature cystic teratoma) and the hyperechoic attenuating component consistent with fat. Additionally, the fat-fluid interface sign was evident on high-resolution ultrasonography images. Serum levels of the tumor marker CA 19-9 were elevated at 92.2 IU/mL, while CA 125 and CEA levels were normal. The patient underwent surgery and the diagnosis was confirmed by histopathological analysis of the surgical specimen. The post-operative recovery period was uneventful and the patient was discharged home after one week. Follow-up imaging at three months did not reveal any features to suggest recurrence. The patient has given written informed consent to publish her case and clinical images.

## Discussion

Teratoma is the most common germ cell tumor of the ovary accounting for approximately 20% of all ovarian tumors [4]. Ovarian teratomas are further classified into sub-categories such as mature cystic teratomas, immature teratomas, and monodermal or highly specialized teratomas (struma ovarii). MCT is the most common among teratomas arising from the ovary and histopathological evidence suggests that at least two well-differentiated mature germ cell layers (ectoderm, mesoderm, endoderm) are required for diagnosis [5]. Ectoderm and mesoderm comprise the most common germ cell layers of teratoma with hair and skin (arising from the ectoderm) and muscle and fat (arising from the mesoderm). Ultrasonography diagnosis of MCT is usually incidental. Although the fat component of MCT appears hyperechoic on ultrasonography, sometimes it poses a diagnostic challenge to differentiate from a complex ovarian cyst /hemorrhagic cyst.

MCT of the ovary is very rarely seen in the postmenopausal age group and has been associated with excessive androgen production [6]. The incidence of signs and morphological appearances of MCT on ultrasonography is described below. The commonest finding of MCT on ultrasonography is intratumoral fat, which demonstrates regional or diffuse high amplitude echoes seen in 93% of cases. Rokitansky nodule or dermoid plug, which is a densely echogenic tubercule projecting into the cystic lumen causing shadowing, is demonstrated in 81-86% of cases. Tuft of hair demonstrating regional or diffuse high amplitude echoes is seen in 65% of cases. Dot-dash sign, which demonstrates hyperechoic dots and lines arising from hairs in different orientations within the imaging plane, is seen in 61% of cases. Chunky calcification /tooth demonstrating regional high amplitude echoes with shadowing is seen in 56% of cases. Comet tail appearance, which is hypoechoic hair strands causing posterior acoustic shadowing, is seen in 12% cases. Fat-fluid and fluid-fluid levels sign demonstrating anechoic sebum layered above the hyperechoic aqueous layer is seen in 8-12% of cases. Tip of the iceberg sign demonstrates a mixture of fat, hair, and cellular debris creating an echogenic focus causing posterior acoustic shadowing to be seen in 4% of cases [7]. The floating balls sign is an uncommon sign of ultrasonography and demonstrates floating hyperechoic fat globules moving with changing position of the patient [8].

## Conclusions

In conclusion, although the diagnosis of ovarian dermoid based on ultrasonography alone is challenging, the radiological signs and sonographic appearances of MCT described in this report help in reliably differentiating MCT from a hemorrhagic lesion or a complex ovarian cyst. Moreover, MCT of the ovary in the postmenopausal age group is an extremely rare entity that may cause virilization and other excessive androgen production-related complications. Careful analysis of the patients presenting complaints is very crucial to reiterate the diagnosis of a benign entity such as MCT to prevent unnecessary testing for tumor markers and advanced imaging, especially in a resource-starved setting. The diagnostic value of elevated CA 19-9 in patients with MCT would be poor when used alone. Furthermore, timely diagnosis and prompt recognition of MCT-related complications on ultrasonography have a prognostic significance.
